# Low spatial frequency laser-induced periodic surface structures in fused silica inscribed by widely tunable femtosecond laser pulses

**DOI:** 10.1038/s41598-022-24771-9

**Published:** 2022-11-23

**Authors:** Marius Navickas, Robertas Grigutis, Vytautas Jukna, Gintaras Tamošauskas, Audrius Dubietis

**Affiliations:** Laser Research Center, Vilnius University, Saulėtekio Avenue 10, 10223 Vilnius, Lithuania

**Keywords:** Optics and photonics, Applied optics

## Abstract

The formation and evolution of laser-induced periodic surface structures in fused silica under irradiation of widely tunable (in the 1–3 $\upmu$m range) linearly polarized femtosecond (200 fs) pulses was studied experimentally. The structures were inscribed in high fluence regime (exceeding the surface ablation threshold for a single pulse) and characterized by using scanning electron microscopy and two dimensional Fourier transform. The results revealed rapid (after irradiation with a few successive pulses) formation of periodic laser-induced periodic surface structures aligned parallel to laser polarization, whose period increases with increasing the inscription wavelength, obeying the $\lambda /n$ law. With further increase of number of pulses, the generated structures gradually reorganize into laser polarization-independent low spatial frequency annular structures associated with formation of the damage crater, which fully established after irradiation with a few tens of successive laser pulses. This particular evolution scenario was observed over the entire wavelength tuning range of incident pulses.

## Introduction

Laser-induced periodic surface structures (LIPSS) spontaneously emerging from irradiation of solid-state materials with repetitive femtosecond laser pulses attract great scientific and technological interest and offer new perspectives for laser material processing^1–8^. These structures arise from permanent material modifications due to complex light-driven material reorganization, whose key features, such as topography, periodicity and orientation, are functions of material properties, irradiation geometry, laser wavelength, polarization state, peak fluence, and number of pulses. According to periodicity, LIPSS are classified into high spatial frequency LIPSS (HSFL) and low spatial frequency LIPSS (LSFL), with characteristic periods of $\sim \lambda /2n$ and $\sim \lambda /n$, respectively, where *n* is the refractive index of material^4^. HSFL and LSFL are commonly observed LIPSS morphologies, although there exist more specific surface topographies, e.g. nanoparticle strings and quasiperiodic microspikes, that emerge upon ultrashort pulse irradiation of a conductive solid^9, 10^.

Many experiments on LIPSS formation were performed in various glasses and in fused silica, in particular, which represents versatile and one of the most relevant optical materials, finding diverse applications in contemporary optical sciences and technology^11^. Transformation of LIPSS morphology from HSFL to LSFL was observed with the increase of number of impinging pulses^12^ and laser fluence^13^, in particular demonstrating the transition from the high spatial frequency perpendicularly oriented nanoripples to the low spatial frequency ripples, parallel or perpendicular to the laser polarization^14^. Formation of more sophisticated forms of material reorganization, such as nanoplillar arrays^15^ and supra-wavelength periodic surface structures, whose periodicity may exceed laser wavelength by several times^16, 17^, were reported so far. Irradiation by successive femtosecond laser pulses in high fluence regime produces polarization-independent low spatial frequency annular structures (LIPASS) at the center of the laser ablation crater^18^. Further studies uncovered the change of surface morphology from HSFL to LSFL and eventually to LIPASS, with increasing the number of pulses^19^. A similar evolution was observed in As$_2$S$_3$ chalcogenide glass^20^ and more recently, in various transparent dielectric materials^21^, attesting the universality of such morphological transition.

Tailoring of LIPSS topography was demonstrated by employing double pulse^22^ and two-color (near-IR fundamental and UV second harmonic)^23, 24^ irradiation, and by using pulse trains with designed pulse delays^15^. The experiments performed using single pulse fluence above laser-induced damage threshold, demonstrate that LSFL are produced without an intermediate step of HSFL formation^25, 26^. However, so far the vast majority of experiments on LIPSS formation in fused silica were performed within a very narrow spectral region, using fundamental harmonics of standard femtosecond Ti:sapphire and Yb-doped laser sources, emitting at around 800 nm and 1.03 $\upmu$m, respectively. Much fewer experimental investigations were performed with UV^23, 24^ and near-IR pulses having longer wavelengths^19^, while only theoretical study was conducted regarding LIPPS formation in the mid-IR^27^.

In this work, we study pulse-by-pulse formation dynamics and evolution of laser-induced periodic structures on the surface of fused silica over a broad wavelength range using widely tunable femtosecond pulses. The experiments were performed in the regime when a single pulse fluence exceeds surface ablation threshold and reveal rapid formation of LSFL with periods obeying the $\lambda /n$ law, which thereafter are reorganized into LIPASS due to formation of the ablation crater.

## Methods

### Experimental

**Figure 1:**
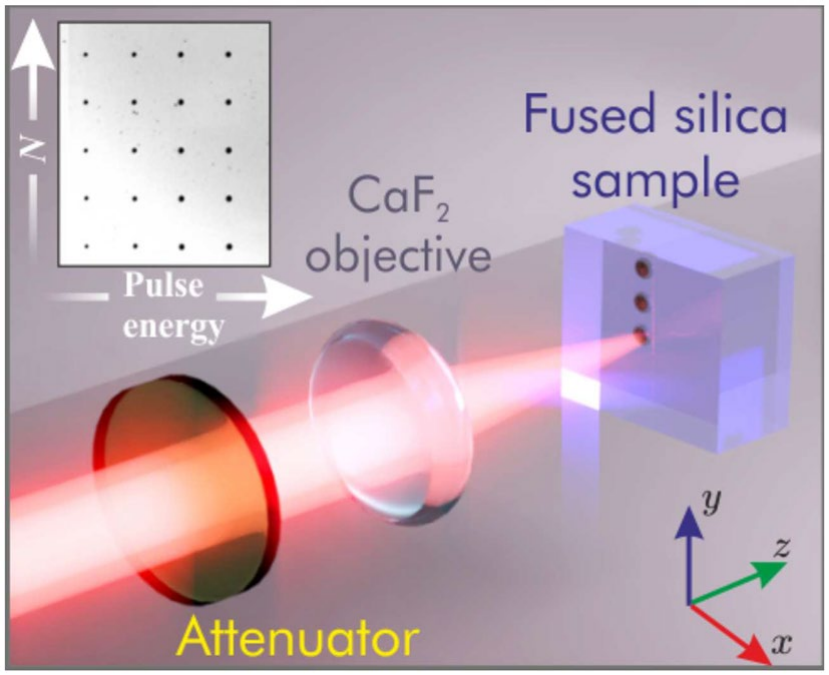
Sketch of the experimental setup employed for LIPSS formation on fused silica. The inset shows optical microscope image of the array of damage structures produced by varying energy and number of successive pulses.

The experiments were performed with 200 fs, 1.32–3.0 $\upmu$m-tunable spatially Gaussian pulses from an optical parametric amplifier (OPA, Orpheus-HP, Light Conversion Ltd.), which was pumped by fundamental harmonic of amplified Yb:KGW laser (Pharos, Light Conversion Ltd.) operating at 10 kHz repetition rate. The experimental setup is sketched in Fig. 1. The linearly polarized OPA beam was focused onto the front face of uncoated UV-grade fused silica sample with a $f=+\, 15$ mm home-made objective consisting of a pair of CaF$_2$ lenses. A supplementary experiment was performed using the fundamental harmonic (1035 nm) pulse directly from the laser; in this case, the laser beam was focused with a $f=+\, 100$ mm lens. The input pulse energy was adjusted using metal-coated variable neutral density filter (NDC-25C-2, Thorlabs Inc.). At each wavelength the optimal energy was set experimentally so as to exceed surface ablation threshold^28^ with a single impinging pulse. More specifically, the operating conditions at each wavelength were chosen to produce the ablation craters of a similar size, setting the input pulse energy approximately 1.5 times above the threshold energy where the ablation crater becomes detectable with an optical microscope (Olympus BX51).

To estimate the peak fluence and intensity, we first calculated the focal spot size using an expression $2w_{0}=\frac{4 M^{2} f \lambda }{\pi (2w)}$, where $\lambda$ is wavelength and $M^2$ is the beam quality parameter that was taken as 1.1 and 1.3 for the laser fundamental harmonic (1035 nm) and the OPA beams, respectively, as specified by the producer. The input beam diameter 2*w* (at the $1/e^2$ intensity level) was measured by a knife-edge method^29^. These experimental parameters for each wavelength are specified in Table 1. Note that fluence and intensity do not show monotonous increase versus wavelength due to the change of multiphoton absorption order, wavelength dependence of ionization rate and inverse bremsstrahlung that provides acceleration of free electrons to initiate the avalanche processes^30^, and eventually, the onset of linear absorption of fused silica for wavelengths longer than 2500 nm.

**Table 1:** Summary of the experimental parameters: $\lambda$ is the pulse central wavelength, $\text {2}w$ is the beam diameter on a focusing lens, $\text {2}w_{0}$ is the estimated diameter of the focal spot, *E* is the pulse energy, *F* is the peak fluence, *I* is the peak intensity and *N* is the number of pulses.

$\lambda$ (nm)	2*w* (mm)	2$w_{0}$ ($\upmu$m)	*E* ($\upmu$J)	*F* (J/cm$^2$)	*I* (TW/cm$^2$)	*N* (pulses)
1035	5.1	28	11	3.6	18.6	10
1500	1.5	25	8	3.3	15.3	10
1700	2.0	21	8	4.6	21.7	10
1900	2.0	24	8	3.5	16.6	10
2500	1.7	37	14	2.6	12.2	12
2900	2.0	36	18	3.5	16.6	12

The fused silica sample was mounted on a motorized three-dimensional translation stage. The LIPSS were produced by varying number of incident pulses using the laser built-in programmable pulse picker and inscription wavelength, keeping a fixed fluence, as specified in Table 1. After each series of laser exposure, the sample was translated to a “fresh” (unexposed) site in the plane perpendicular to beam propagation direction (in the $x{-}y$ plane), so recording an entire matrix of structures, as shown in the inset of Fig. 1. Thereafter the recorded LIPSS were imaged and characterized with a scanning electron microscope (SEM, Prisma E, Thermo Fisher Scientific).

## Results and discussion

**Figure 2:**
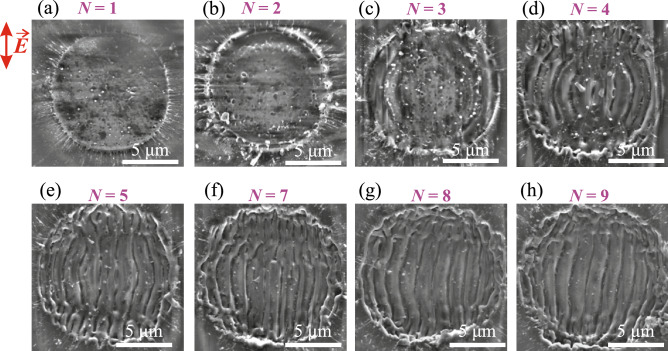
SEM images of fused silica surface after irradiation with different number of linearly polarized 200 fs, $1.7~\upmu$m pulses with a fixed fluence of 4.6 J/cm$^2$. The red arrow indicates polarization direction of the incident laser pulse.

The number of laser pulses irradiating the same area is one of the essential parameters governing the overall LIPSS formation dynamics, morphology and periodicity, see e.g.^12^. Fig. 2 displays SEM images of the irradiated area showing formation and pulse-by-pulse evolution of LIPSS in fused silica produced with OPA pulses having a central wavelength of 1700 nm (see the experimental parameters specified in Table 1). The first impinging pulse ($N=1$, Fig. 2a) melts and ablates the material, producing a sharply defined shallow crater with diameter of $\sim 10~\upmu \text {m}$. The ablation crater has no clear features apart a number of scattered nanoparticles which may act as an initiators of the nanograting formation via laser field scatter. The second pulse ($N=2$, Fig. 2b) only slightly deepens, enlarges the crater and slight organization of the nanoparticles on a ring is evident. As the third pulse ($N=3$, Fig. 2c) irradiates the previously formed crater, the interference of the incident radiation field and electromagnetic waves scattered on a rough surface leads to spatially modulated impinging electric field^14^. As a result, the modulated deposition of energy occurs via generation of periodic density of quasi-free electrons through multiphoton absorption (13-photon absorption, assuming fused silica bandgap of 9.0 eV and incident photon energy of 0.73 eV) that is further amplified by inverse bremsstrahlung effect and avalanche ionization. This in turn promotes the generation of spatially modulated electronic defect states that modify the lattice system and eventually lead to surface modulation^14, 27^. After the arrival of fourth successive pulse, surface modulation takes a more definite character with feature size of the order of laser wavelength ($N=4$, Fig. 2d). Fully developed regular LSFL pattern with orientation parallel to the polarization of the laser pulse is established after five laser pulses ($N=5$, Fig. 2e), which thereafter does not change significantly with the arrival of more successive laser pulses, as attested by SEM images in Figs. 2f,g,h.

**Figure 3:**
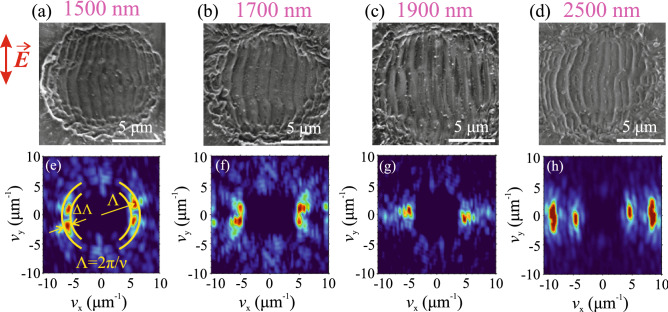
SEM images of LSFL in fused silica, inscribed with OPA pulses having central wavelengths of (**a**) 1500 nm, (**b**) 1700 nm, (**c**) 1900 nm and (**d**) 2500 nm pulses. (**e–h**) The corresponding spatial frequency spectra retrieved by 2D-FFT. The red arrow indicates polarization direction of the incident laser pulse.

The observed LSFL formation without an intermediate step of HSFL is in line with earlier experimental findings reported in high fluence regime using fundamental harmonics (1025 and 1030 nm) of femtosecond Yb lasers^25, 26^. Our experiments performed with inscription wavelengths of 1035, 1500, 1900, 2500 and 2900 nm uncovered essentially identical scenario of LSFL formation as reported above, despite the fact that the underlying effects contributing to free electron generation, such as multiphoton absorption and inverse Bremsstrahlung exhibit strong wavelength dependence. Figure 3a–d present examples of LSFL patterns produced with incident pulses having central wavelengths of 1500, 1700, 1900 and 2500 nm under experimental conditions listed in Table 1.

**Figure 4:**
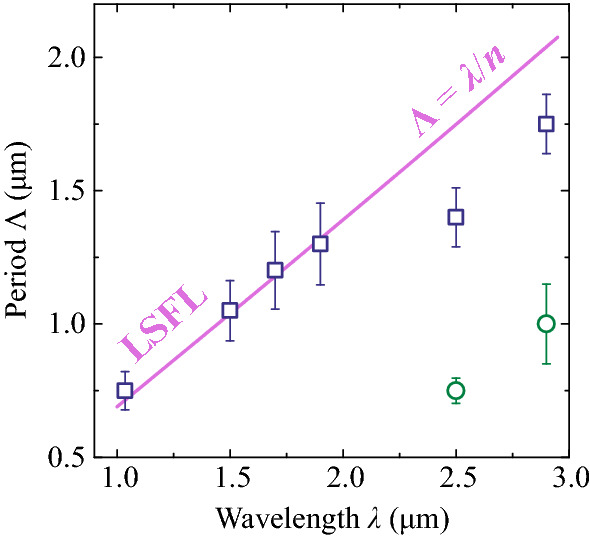
LSFL period as a function of inscription wavelength. Pairs of data points at 2500 and 2900 nm illustrate formation of double periodic structures, see text for details.

In what follows, we analyzed the periodicity of these structures by applying two dimensional fast Fourier transform (2D-FFT) on SEM images. Figure 3e–h show the respective spatial frequency spectra, which consist of relatively compact ridges positioned along $\nu _x$ axis, confirming parallel orientation of the LSFL with respect to the polarization of incident laser pulse. The estimated LSFL periods versus the inscription wavelength are summarized in Fig. 4. The LSFL periods were calculated as $\Lambda =2\pi /\nu$, taking the peak value of spectral intensity from the central slice of the ridge in the ($\nu _{\textrm{x}}$, $\nu _{\textrm{y}}$) space, while the error bars were estimated as full widths at half maximum of the same slice, as schematically illustrated in Fig. 3e.

The estimated LSFL period of $750 \pm 72$ nm as inscribed by incident fundamental laser harmonic (1035 nm) pulses is very close to previously reported value of 735 nm, obtained with a similar laser wavelength (1025 nm) and similar fluence of incident pulses^26^. The overall wavelength dependence demonstrates that the LSFL period increases with the increase of inscription wavelength, and in the 1–2 $\upmu$m range the estimated LSFL periods nicely follow the $\lambda /n$ trend, which is depicted by a solid line. Interestingly, the spatial frequency spectrum of LSFL at 2500 nm (Fig. 3h) reveals four ridges, suggesting two distinct periods of 1400 nm and 750 nm, which both are plotted in Fig. 4. A closer inspection of Fig. 3d reveals that the smaller period (the higher spatial frequency) is attributed to clearly dual sub-structure of individual ripples, and to some extent, to apparently more densely-packed structures located at the margins of irradiated area. A very similar double-periodic feature was also found in LSFL at 2900 nm (the SEM image is not shown, but measured LSFL periods are depicted in Fig. 4). In our opinion, formation of these specific structures is related to the onset of linear absorption in fused silica, which first contributes to the ablation process and therefore alters the LSFL formation and its periodicity.

**Figure 5:**
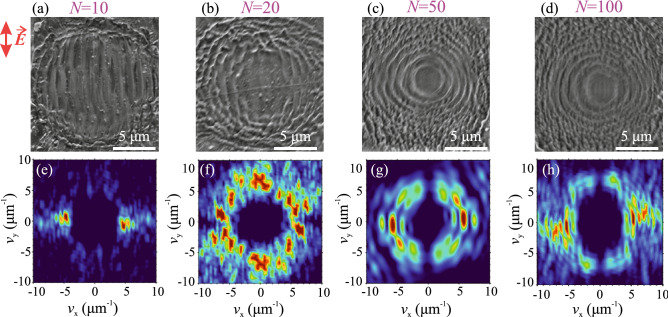
(**a–d**) SEM images of surface structures inscribed with 10, 20, 50 and 100 successive pulses. The pulsewidth is 200 fs, the central wavelength is 1900 nm, the peak fluence is 3.5 J/cm$^2$. (**e–h**) The corresponding spatial frequency spectra retrieved by 2D-FFT.

Finally, we investigated the post-evolution of LSFL by further increasing the number of successive laser pulses. Fi. 5a–d present SEM images of surface structures inscribed with 10, 20, 50 and 100 successive pulses having a central wavelength of 1900 nm. The images show gradual reorganization of periodic vertically aligned ripples into annular (laser polarization-independent) periodic fringes, attesting the change of LIPSS morphology from LSFL to LIPASS, whose formation is attributed to the interference between the incident laser field and that reflected from the crater walls^18^. Figure 5e–h present the respective spatial frequency spectra, which confirm the transition from linear to annular topography of the induced structures. The spatial frequency spectra also indicate a decrease of structure period from 1.3 to 1.0 $\upmu$m, when the number of successive laser pulses increases from 10 to 100. Note that the apparent period of annular rings is a projection of the surface fringe onto the crater cone walls, so is a function of crater width and depth. The crater dimensions (depth of $8~\upmu$m and width of 12 $\upmu$m) were estimated by inspection of the structure using an optical profilometer (Sensofar PL$\mu$ 2300), yielding the crater cone angle of $\sim ~72^\circ$ and so the annular fringe period of 0.8 $\upmu$m, which is close to the experimental value retrieved from 2D-FFT. No further noticeable change of the structure topography, as well as its period was detected after irradiation with 50 laser pulses. This suggests that the ablation crater is already fully formed, or at least its further development slows down considerably due to change of irradiation geometry resulting in a decrease of laser fluence, as the incident pulse energy becomes distributed over entire surface area of cone-shaped ablation crater, compared with irradiation of almost flat surface producing LSFL. Finally, we verified that LSFL evolve into LIPASS using shorter (1035 nm) as well as longer (2500 nm) inscription wavelengths, suggesting essentially identical post-evolution scenario of LSFL.

## Conclusions

In conclusion, the experimental investigation of pulse-by-pulse inscription dynamics of laser-induced periodic structures on the surface of fused silica with femtosecond pulses in high fluence regime uncovered the evolution scenario which is valid for a wide range (1–3 $\upmu$m) of inscription wavelengths. In particular, our experiments demonstrated rapid emergence of LSFL aligned parallel to laser polarization, which thereafter gradually reorganize into laser polarization-independent LIPASS. Our findings underline the role of laser wavelength aiming at inscription of LIPSS with desired and well-controlled periodicity. We expect that our results could be helpful also for validating the existing numerical models, especially for simulating the dynamics of periodic surface structures inscribed with currently available widely tunable ultrafast light sources based on optical parametric amplification.

## Data Availability

The datasets used and/or analysed during the current study available from the corresponding author on reasonable request.
